# Overweight status of the primary caregivers of orphan and vulnerable children in 3 Southern African countries: a cross sectional study

**DOI:** 10.1186/s12889-015-2061-2

**Published:** 2015-08-07

**Authors:** Mariano Kanamori, Olivia Carter-Pokras, Sangeetha Madhavan, Robert Feldman, Xin He, Sunmin Lee

**Affiliations:** Center for Research on U.S. Latino HIV/AIDS and Drug Abuse, Florida International University, 11200 SW 8th Street. AHC5 422, Miami, FL 33199 USA; Department of Epidemiology and Biostatistics, University of Maryland College Park School of Public Health, 2234G SPH Bldg, College Park, MD 20742 USA; African American Studies Department, 1119 Taliaferro Hall, College Park, MD 20742 USA; Department of Behavioral and Community Health, School of Public Health University of Maryland, College Park, MD 20742 USA

**Keywords:** Orphans, Overweight, Africa South of the Sahara

## Abstract

**Background:**

Africa is facing a nutritional transition where underweight and overweight coexist. Although the majority of programs for orphan and vulnerable children (OVC) focus on undernourishment, the association between OVC primary caregiving and the caregivers’ overweight status remains unclear. We investigated the association between OVC primary caregiving status with women’s overweight status in Namibia, Swaziland and Zambia.

**Methods:**

Demographic Health Survey (DHS) cross-sectional data collected during 2006–2007 were analyzed using weighted marginal means and logistic regressions. We analyzed data from 20–49 year old women in Namibia (*N* 6638), Swaziland (*N* 2875), and Zambia (*N* 4497.)

**Results:**

The overweight prevalence of the primary caregivers of OVC ranged from 27.0 % (Namibia) to 61.3 % (Swaziland). In Namibia, OVC primary caregivers were just as likely or even less likely to be overweight than other primary caregivers. In Swaziland and Zambia, OVC primary caregivers were just as likely or more likely to be overweight than other primary caregivers. In Swaziland and Zambia, OVC primary caregivers were more likely to be overweight than non-primary caregivers living with OVC (Swaziland AOR = 1.56, Zambia AOR = 2.62) and non-primary caregivers not living with OVC (Swaziland AOR = 1.92, Zambia AOR = 1.94). Namibian OVC caregivers were less likely to be overweight than non-caregivers not living with an OVC only in certain age groups (21–29 and 41–49 years old).

**Conclusions:**

African public health systems/OVC programs may face an overweight epidemic alongside existing HIV/AIDS, tuberculosis and malaria epidemics. Future studies/interventions to curb overweight should consider OVC caregiving status and address country-level differences.

## Background

Primary caregivers worldwide are facing the dilemma of maintaining their own health while addressing the physical and emotional needs of family members [1]. Primary caregiving of children is particularly challenging in Africa, where the Acquired Immune Deficiency Syndrome and the Human Immunodeficiency Virus (HIV/AIDS) pandemic has increased the number of orphan and vulnerable children (OVC) in need of care. More than four-fifths of all OVCs (nearly 12 million) live in Africa. The prevalence of OVC varies widely across countries and across different population sub-groups—with countries and sub-regions with a higher prevalence of HIV having a higher prevalence of OVC as well [2].

Rates of overweight and obesity are high and rising in Africa, particularly among women, and are a cause for concern [3]. Equally relevant is that over- and under-nutrition often co-exists in the same household, particularly with adults being overweight and children underweight. Due to frequent population flows between urban and rural areas, lifestyle habits may be changing with uptake of unhealthier diets and sedentary lifestyles that contribute to obesity occurring even in rural sub-Saharan African settings [3]. It has been suggested that the presence of under-nutrition and overweight and obesity among adolescent girls is due to changes in traditional diets, dependence on processed foods and insufficient local food production [4]. Overweight is traditionally desirable among African women, and thought to reflect success and not having HIV/AIDS.

This study focuses on overweight problems in three southern sub-Saharan African countries because the BMI of women between the ages of 15–49 years is much higher in southern Africa than in other African regions [5]. The impact of caring for OVC on the primary caregivers’ nutrition among 20–49 year old women in Africa remains inadequately understood due to the limited research conducted to date, which has had small sample sizes, lacked appropriate comparison groups, and was predominantly qualitative [6]. In addition, the majority of studies on caregiving and nutritional outcomes focused on adults 60 years and older caring for children and grandchildren [7–9]. The limited number of studies on the impact of caring for OVC on primary caregivers’ BMI in Africa may be due to limited access to relevant national data and researchers in the area [10, 11]. Furthermore, research addressing women’s nutrition in Africa has focused primarily on rural areas or specific regions of a country [9, 10]. Focusing on the overweight status of OVC primary caregivers in Africa is important because child caregiving could serve as an additional stressor and lead to psychological distress that may increase cortisol and catecholamine, lead to unhealthy behaviors (e.g., eating poorly), and result in increased BMI [12]. Women caring for OVC are experiencing more severe economic and social problems (e.g., discrimination, stigma due to HIV/AIDS and lower income) than other women from their own towns [13, 14].

To the best of our knowledge, no previously published studies have investigated the association between overweight status and reproductive age OVC primary caregivers in sub-Saharan Africa. In order to address this research gap, this cross-sectional study investigates the association between caring for OVC with women’s overweight status in Namibia, Swaziland and Zambia. Four mutually exclusive child caregiving groups were included. Two groups included primary caregivers of a child: a) primary caregivers of OVC and b) primary caregivers of only non-OVC. Two additional child caregiving groups included women who were non-primary caregivers of a child: c) non-primary caregivers of a child who were living with an OVC and d) non-primary caregivers of a child who were not living with an OVC. The following research objectives guided this study. First, determine whether the prevalence of overweight is higher than the prevalence of underweight among OVC primary caregivers. Second, determine whether the OVC primary caregivers’ mean body mass index (BMI) were different than the mean BMI of women from the other three child caregiving groups within countries. Third, determine whether the OVC primary caregivers’ mean BMI varied significantly by country. Fourth, identify significant associations between caring for OVC and women’s overweight status in each country. Fifth, identify whether socio-demographic and household characteristics (e.g., age, work status, number of household members, household wealth) modified the effect of the association between OVC primary caregiving status and women’s overweight status in each country.

## Methods

This cross-sectional study was based on secondary analyses of the most current available data from the Demographic Health Survey (DHS) from Namibia (2006–2007), Swaziland (2006–2007), and Zambia (2007). In order to have a representative sample of the population, DHS surveys involved two stages of sampling. The first stage was based on an up-to-date sampling frame, i.e., a list of small administrative units with defined boundaries and known population size, usually census enumeration areas (EAs). Around 300–500 of these EAs were selected from the sampling frame list with probability proportional to population size [15]. After the EAs (i.e., clusters) were selected, a household listing operation was implemented. This involved sending a small team of field workers (usually 2 people) to each selected EA to locate the boundaries, draw a sketch map, and prepare a list with the name of the head and the address or location of each household. In the selected households, all women of reproductive age (15–49) were eligible for an individual interview [15]. The training consisted of classroom lectures, mock interviews, and practical interviews in the field. Based on the performance during training, participants were recruited to work as supervisors, field editors, enumerators, and data entry personnel [15].

DHS survey teams were assigned sample areas taking into account languages spoken and other requirements and the need to ensure that the travel times per team were minimized as much as possible [15]. If an interview was not completed on the first visit, further attempts were made with the sampled household or respondent (up to three times and over three different days) before classifying the case as non-response. The subsequent contacts were scheduled at times when the respondent was more likely to be at home. There was no replacement for a household or an individual that refused to be interviewed or was otherwise classified as non-response. Eligible women response rates in every country and region included in this study reached at least 94 %. The Macro Institutional Review Board approved every DHS survey. Participants provided consent for participation.

For the purposes of this paper, the country inclusion criteria were: located in Southern Africa (the region most heavily affected by the HIV epidemic and with the highest prevalence of overweight and number of wealthiest countries on the continent), HIV prevalence of at least 5 % or orphan prevalence of at least 8 % among 0–17 year old children (i.e., one or both biological parents have died), had less than 20 % missing BMI data, belong to the Southern African Development Community, had economies linked to South Africa, and had available DHS data for primary caregiving status and women’s anthropometry.

### Participants’ inclusion and exclusion criteria

*Inclusion criteria:* women were included if they slept in the household the night before the survey (de facto household residence) and if they were 20–49 years old since many 15–19 year old women would still be in the adolescent growth spurt period and some of them still in puberty.

*Exclusion criteria:* women were excluded from the analyses if they were pregnant and three months or less postpartum women to avoid the impact of the fetus and lactation on the BMI [16], and/or if their BMI was less than 12.0 or BMI greater than 60.0 because these might be cases of extreme anthropometric measures or resulting from data errors [17].

### Measures

#### Dependent variable

The dependent variable was women’s BMI also known as the Quetlet index. BMI was defined as weight in kilograms divided by height squared in meters (kg/m^2^). BMI was analyzed as a continuous variable (research questions 1 and 2) and as a categorical variable (research questions 3 and 4) using two categories: normal weight (18.5 ≤ BMI < 25.0) and overweight (BMI ≥ 25.0). BMI has been widely used as an anthropometric indicator of health, especially for nutrition-related disease among adult women from Sub-Saharan countries and other regions [16]. Weight was measured using a solar-powered scale (Uniscale) with an accuracy of ± 100 g [16]. Height was measured to the nearest 0.1 cm with an adjustable wooden measuring board (Shorr Height boards).

### Independent variable

***Primary caregivers of a child*** were women who lived with a biological child under the age of 18 and/or who were primary caregivers of a non-biological child under the age of 18 [assessed using the question “(Besides your own child/children), are you the primary caregiver for any children under the age of 18?”

***The OVC primary caregiving status of a woman*** was based on the question: “How many orphans and vulnerable children live in your household?” This study used the DHS definition for OVC: children with one or both parents deceased (orphans); and vulnerable children who a) had a chronically ill parent (sick for more than 3 consecutive months during the past 12 months) or b) lived with an adult who was chronically ill or died during the past 12 months. There were four mutually exclusive caregiving categories. ***Primary caregivers of an OVC*** provided care to one or more OVC (biological or non-biological) and included women who were primary caregivers of both OVC and non-OVC children. ***Primary caregivers of a non-OVC*** provided care to only non-OVC children (biological or non-biological). ***Non-primary caregivers living with an OVC*** lived with one or more OVC. ***Non-primary caregivers not living with an OVC*** did not live with an OVC.

### Potential confounders or effect modifiers

*The following socio-demographic and household characteristic variables were considered as potential confounders or effect modifiers:****presence of a child 5 years old or younger living in the house*** (yes, no), **w*omen’s marital status*** (married or living together, divorced, widowed, never married, and not living together); ***women’s age*** (20–29, 30–39 and 40–49 years old); ***women’s education*** (no schooling, primary school, secondary education, and higher education); ***region of residence*** (rural, urban); ***number of children ever born*** (0, 1, 2, and *≥*3 births); ***women’s relationship with the household head*** (head, wife, daughter, other); ***sex of the household head*** (male, female); ***number of household members****(less than 3, 4 to 6, ≥7);****number of 18–49 year old women in the household****(1, 2, 3, ≥4);****number of 18–49 year old men in the household****(0, 1, ≥2); and,****women’s work status*** (not working, working in agriculture, and working in any field other than agriculture). We also measured ***household wealth*** using the ***Absolute Wealth Index****(AWI),* a continuous measurement ranging from 0 (no modern goods) to 12+ (all modern comforts, for example flushing toilet, electricity, means of transportation, telephone, etc.) For the purpose of this study, we used a categorical AWI: poorest (0–1, reference group), poorer (2–3), medium (4–5), wealthier (6–7), and wealthiest (8+) [17, 18].

### Analysis

Statistical analyses included data screening to check for outliers and errors as well as descriptive statistics for continuous and discrete variables. Unadjusted and adjusted logistic regression models were used to determine potential confounders. A variable was included as a confounder if the adjusted odds ratio (OR) varied by more than 10 % than the unadjusted OR. Collinearity between each pair of independent variables was tested by using Phi coefficients when both variables were dichotomous and Cramer’s V for variables with three or more levels (a value ≥ .60 showed substantial collinearity and one variable was removed from the analysis). Multicollinearity among the independent variable and all potential confounders entered in the model was tested using the tolerance value and the variance inflation factor (VIF). A tolerance value less than 0.1 was considered as an indication for a serious collinearity problem and a VIF greater than 10 was also considered as a cause for concern and one variable was removed from the analysis [19].

The estimation of marginal means used analytic weights included in the data set to correct for over-sampling and variations in survey response rates by region. Three logistic regression models were performed for each country. In order to report the likelihood for OVC primary caregivers to be overweight as compared to women from the other three caregiving groups, each model included a different OVC primary caregiving reference group. The odds ratio between the OVC primary caregiving category and each of the other three caregiving group categories were presented separately. These logistic regression analyses controlled for potential confounders and interactions, and did not include sampling weights as recommended in the guide for DHS statistics by Rutstein and Rojas (2006). To assess the presence of an effect modifier, one interaction term between the variable OVC primary caregiving status and each potential effect modifier was included in each logistic regression model. If this interaction term was significant, further stratified analyses were performed. Significant associations were assessed using 95 % confidence intervals and p-values (α ≤ 0.05). Analyses were performed using SPSS® 19.

## Results

### Missing data

Missing data were addressed using the listwise deletion approach, also known as complete case analysis. This technique omitted those cases with missing data. Although listwise deletion could result in a substantial decrease in the sample size available for analysis, it does have important advantages. In particular, under the assumption that data are missing completely at random, it leads to unbiased parameter estimates. The assumption of missing completely at random was checked using the SPSS missing value analyses procedures including: univariate statistics (number of non-missing values, mean, standard deviation, number of missing values, and number of extreme values); t-tests with groups formed by indicator variables; cross tabulation of categorical and indicator variables; pattern analyses including tabulated cases grouped by missing value patterns; and the Little’s MCAR test with EM results. Missing data for BMI was: Namibia 2.9 %, Swaziland 2.9 % and Zambia 1.2 %. Missing data for the presence of OVC at home was: Namibia 0.0 %, Swaziland 12.5 % and Zambia 0.0 %. These procedures were performed with variables that had more than 10 % of missing information (only for the presence of an OVC in the household in Swaziland).

Our analyses showed that if a respondent was from a rural area in Swaziland, a measure regarding the presence of an OVC in the household was more likely to be missing (Table 1). For example, 16.3 % of missing data for rural areas and 5.2 % for urban areas were found for the variable “presence of an OVC in the household”. There did not seem to be other missing data discrepancies for other socio-demographic characteristics. Also, it appeared that the mean Absolute Wealth Index and the mean BMI were similar between the whole dataset (including missing and not missing cases) and the dataset only including missing cases for the presence of an OVC in the household. In order to confirm the data were missing at random, Little’s MCAR tests was performed. Because the p-value was above 0.05, it was concluded that the assumption was not violated and it was appropriate to listwise delete cases with missing data for the presence of an OVC in the household in Swaziland.

**Table 1:** Comparison of missing percentages by socio-demographic characteristics and mean of women’s age, mean of Absolute Wealth Index, and mean of BMI by missing and non-missing cases

	Swaziland Missing data for the presence of OVC at home % of missing cases
Region	
Urban	5.2
Rural	16.3
Women’s education	
No education	11.8
Primary education	11.9
Secondary education	13.5
Higher education	10.4
Marital status	
Never married	14.0
Married	12.0
Widowed	9.4
Divorced	10.0
Work status	
Not working	13.4
Agriculture	8.1
Other than agriculture	12.2
Women age (mean)	
Missing and non-missing cases	32.04
Only missing cases	31.79
Household Wealth Index (mean)	
Missing and non-missing cases	6.21
Only missing cases	5.86
BMI (mean)	
Missing and non-missing cases	29.53
Only missing cases	30.07
Little’s MCAR Test	Chi-square = 5.37, DF = 3, *p*-value = 0.15

### Sample characteristics

Analyses were performed using data from 20–49 year old women in Namibia (*n* = 6638), Swaziland (*n* = 2875), and Zambia (*n* = 4497). The percentages of OVC primary caregiving were similar among countries and ranged from 26.6 % in Namibia to 28.6 % in Zambia. A higher proportion of women had secondary education in Namibia and Swaziland than in Zambia. The majority of women in Swaziland lived in rural areas; and, around half of Namibian and Zambian women resided in rural areas. While the majority of Namibian and Zambian women had normal weight; the majority of women in Swaziland were overweight (Table 2). In all countries, more than half of women were living with a child less than 6 years old.

**Table 2:** Socio-demographic characteristics of the women by country

	Namibia (*N*=6638)						Swaziland (*N*=2875)						Zambia (*N*=4497)					
	OVC primary caregiver	Non-OVC primary caregiver	Non-primary caregiver living with OVC	Non-primary caregiver not living with OVC	Total	*p*-value^*^	OVC primary caregiver	Non-OVC primary caregiver	Non-primary caregiver living with OVC	Non-primary caregiver not living with OVC	Total	*p*-value^*^	OVC primary caregiver	Non-OVC primary caregiver	Non-primary caregiver living with OVC	Non-primary caregiver not living with OVC	Total	*p*-value^*^
Nutritional Status^a^, n (%)																		
-Underweight	234	313	101	166	814	<0.001	13	23	4	12	52	<0.001	95	228	26	32	381	<0.001
	(13.0 %)	(10.8 %)	(14.9 %)	(13.0 %)	(12.3 %)		(1.7 %)	(1.4 %)	(3.3 %)	(3.6 %)	(1.8 %)		(7.4 %)	(8.9 %)	(10.6 %)	(8.0 %)	(8.5 %)	
-Normal weight	964	1,440	425	725	3,554		291	569	65	187	1,112		850	1813	175	284	3122	
	(53.6 %)	(49.9 %)	(62.8 %)	(56.8 %)	(53.5 %)		(37.0 %)	(34.9 %)	(53.3 %)	(55.5 %)	(38.7 %)		(65.8 %)	(70.8 %)	(71.1 %)	(71.0 %)	(69.4 %)	
-Overweight	600	1,133	151	386	2,270		482	1,038	53	138	1,711		347	518	45	84	994	
	(33.4 %)	(39.3 %)	(22.3 %)	(30.2 %)	(34.2 %)		(61.3 %)	(63.7 %)	(43.4 %)	(40.9 %)	(59.5 %)		(26.9 %)	(20.2 %)	(18.3 %)	(21.0 %)	(22.1 %)	
Women’s Age in years, Mean (Standard Deviation)																		
	33.69	33.53	28.63	28.86		<0.001	33.20	32.98	25.38	27.15		<0.001	32.56	32.17	27.12	28.69		<0.001
	(8.1)	(7.9)	(8.4)	(8.4)		0.0010	(8.6)	(8.3)	(6.9)	(8.2)			(8.0)	(8.2)	(7.9)	(8.8)		
Women’s Education, n(%)																		
-No Education	168	334	46	72	620	<0.001	88	161	7	40	296	<0.001	126	355	20	42	543	<0.001
	(9.3 %)	(11.6 %)	(6.8 %)	(5.6 %)	(9.3 %)		(11.2 %)	(9.9 %)	(5.7 %)	(11.9 %)	(10.3 %)		(9.8 %)	(13.9 %)	(8.1 %)	(10.5 %)	(12.1 %)	
-Primary Education	609	866	142	190	1,807		278	502	24	82	886		689	1,509	74	128	2,400	
	(33.9 %)	(30.0 %)	(21.0 %)	(14.9 %)	(27.2 %)		(35.4 %)	(30.8 %)	(19.7 %)	(24.3 %)	(30.8 %)		(53.3 %)	(59.0 %)	(30.1 %)	(32.0 %)	(53.4 %)	
-Secondary Education	921	1,499	446	876	3,742		374	765	74	160	1,373		373	587	110	173	1,243	
	(51.2 %)	(51.9 %)	(65.9 %)	(68.6 %)	(56.4 %)		(47.6 %)	(46.9 %)	(60.7 %)	(47.5 %)	(47.8 %)		(28.9 %)	(22.9 %)	(44.7 %)	(43.2 %)	(27.6 %)	
-Higher Education	100	187	43	139	469		46	202	17	55	320		104	108	42	57	311	
	(5.6 %)	(6.5 %)	(6.4 %)	(10.9 %)	(7.1 %)		(5.9 %)	(12.4 %)	(13.9 %)	(16.3 %)	(11.1 %)		(8.0 %)	(4.2 %)	(17.1 %)	(14.2 %)	(6.9 %)	
Marital Status, n(%)																		
-Never Married	738	995	508	871	3,112	<0.001	224	459	95	230	1,008	<0.001	84	171	147	190	592	<0.001
	(41.1 %)	(34.5 %)	(75.0 %)	(68.3 %)	(46.9 %)		(28.5 %)	(28.2 %)	(77.9 %)	(68.2 %)	(35.1 %)		(6.5 %)	(6.7 %)	(59.8 %)	(47.5 %)	(13.2 %)	
-Married	928	1741	146	373	3,188		448	1,045	23	98	1,614		993	2,023	67	158	3,241	
	(51.7 %)	(60.3 %)	(21.6 %)	(29.2 %)	(48.0 %)		(57.0 %)	(64.1 %)	(18.9 %)	(29.1 %)	(56.1 %)		(76.9 %)	(79.1 %)	(27.2 %)	(39.5 %)	(72.1 %)	
-Widowed	102	108	16	23	249		107	117	3	9	236		110	161	7	15	293	
	(5.7 %)	(3.7 %)	(2.4 %)	(1.8 %)	(3.8 %)		(13.6 %)	(7.2 %)	(2.5 %)	(2.7 %)	(8.2 %)		(8.5 %)	(6.3 %)	(2.8 %)	(3.8 %)	(6.5 %)	
-Divorced	28	42	7	9	86		7	9	1	0	17		105	204	25	37	371	
	(1.6 %)	(1.5 %)	(1.0 %)	(0.7 %)	(1.3 %)		(0.9 %)	(0.6 %)	(0.8 %)	(0.0 %)	(0.6 %)		(8.1 %)	(8.0 %)	(10.2 %)	(9.2 %)	(8.2 %)	
Work Status^b^, n(%)																		
-Not Working	764	1,074	266	476	2,580	<0.001	366	630	73	156	1,225	<0.001	412	889	105	175	1,581	<0.001
	(42.8 %)	(37.4 %)	(39.7 %)	(37.7 %)	(39.2 %)		(46.6 %)	(38.7 %)	(60.3 %)	(46.4 %)	(42.7 %)		(32.0 %)	(34.8 %)	(43.2 %)	(43.8 %)	(35.2 %)	
-Working in Agriculture	264	298	63	59	684		45	100	6	19	170		326	884	28	76	1,314	
	(14.8 %)	(10.4 %)	(9.4 %)	(4.7 %)	(10.4 %)		(5.7 %)	(6.1 %)	(5.0 %)	(5.7 %)	(5.9 %)		(25.3 %)	(34.6 %)	(11.5 %)	(19.0 %)	(29.3 %)	
-Working other than Agriculture	757	1,497	341	728	3,323		374	900	42	161	1,477		551	783	110	149	1,593	
	(42.4 %)	(52.2 %)	(50.9 %)	(57.6 %)	(50.4 %)		(47.6 %)	(55.2 %)	(34.7 %)	(47.9 %)	(51.4 %)		(42.7 %)	(30.6 %)	(45.3 %)	(37.2 %)	(35.5 %)	
Region of Residence, n(%)																		
-Urban	544	1,494	258	818	3,114	<0.001	130	696	31	196	1,053	<0.001	699	906	181	232	2,018	<0.001
	(30.3 %)	(51.8 %)	(38.1 %)	(64.1 %)	(46.9 %)		(16.5 %)	(42.7 %)	(25.4 %)	(58.2 %)	(36.6 %)		(54.1 %)	(35.4 %)	(73.6 %)	(58.0 %)	(44.9 %)	
-Rural	1,254	1,392	419	459	3,524		656	934	91	141	1,822		593	1,653	65	168	2,479	
	(69.7 %)	(48.2 %)	(61.9 %)	(35.9 %)	(53.1 %)		(83.5 %)	(57.3 %)	(74.6 %)	(41.8 %)	(63.4 %)		(45.9 %)	(64.6 %)	(26.4 %)	(42.0 %)	(55.1 %)	
Religion, n(%)																		
-Protestant	1,364	2,160	512	1,020	5,056	0.009	108	310	30	59	507	0.009	1,042	2,001	198	309	3,550	0.611
	(76.1 %)	(75.0 %)	(75.9 %)	(79.9 %)	(76.3 %)		(13.8 %)	(19.0 %)	(24.6 %)	(17.5 %)	(17.6 %)		(80.8 %)	(78.3 %)	(80.5 %)	(77.4 %)	(79.0 %)	
-Roman Catholic	410	665	152	236	1,463		2	9	0	3	14		225	510	44	83	862	
	(22.9 %)	(23.1 %)	(22.5 %)	(18.5 %)	(22.1 %)		(0.3 %)	(0.6 %)	(0.0 %)	(0.9 %)	(0.5 %)		(17.4 %)	(20.0 %)	(17.9 %)	(20.8 %)	(19.2 %)	
-Other/No Religion	19	55	11	21	106		675	1,311	92	275	2,353		23	45	4	7	79	
	(1.1 %)	(1.9 %)	(1.6 %)	(1.6 %)	(1.6 %)		(86.0 %)	(80.4 %)	(75.4 %)	(81.6 %)	(81.9 %)		(1.8 %)	(1.8 %)	(1.6 %)	(1.8 %)	(1.8 %)	
Children ≤ 5 years in Household, n(%)																		
-No	442	831	399	867	2539	<0.001	178	607	74	227	1,086	<0.001	292	490	159	275	1,216	<0.001
	(24.6 %)	(28.8 %)	(58.9 %)	(67.9 %)	(38.2 %)		(22.6 %)	(37.2 %)	(60.7 %)	(67.4 %)	(37.8 %)		(22.6 %)	(19.1 %)	(64.6 %)	(68.8 %)	(27.0 %)	
-Yes	1356	2055	278	410	4099		608	1023	48	110	1789		1,000	2,069	87	125	3,281	
	(75.4 %)	(71.2 %)	(41.1 %)	(32.1 %)	(61.8 %)		(77.4 %)	(62.8 %)	(39.3 %)	(32.6 %)	(62.2 %)		(77.4 %)	(80.9 %)	(35.4 %)	(31.2 %)	(73.0 %)	
Household Wealth, n(%)																		
-Poorest	369	478	120	184	1,151	<0.001	82	115	10	25	232	<0.001	321	713	43	74	1,151	<0.001
	(20.5 %)	(16.6 %)	(17.7 %)	(14.4 %)	(17.3 %)		(10.4 %)	(7.1 %)	(8.2 %)	(7.4 %)	(8.1 %)		(24.8 %)	(27.9 %)	(17.5 %)	(18.5 %)	(25.6 %)	
-Poorer	522	514	197	172	1,405		138	209	14	37	398		289	853	36	85	1,263	
	(29.0 %)	(17.8 %)	(29.1 %)	(13.5 %)	(21.2 %)		(17.6 %)	(12.8 %)	(11.5 %)	(11.0 %)	(13.8 %)		(22.4 %)	(33.3 %)	(14.6 %)	(21.2 %)	(28.1 %)	
-Medium	253	393	78	170	894		241	419	27	66	753		201	357	34	53	645	
	(14.1 %)	(13.6 %)	(11.5 %)	(13.3 %)	(13.5 %)		(30.7 %)	(25.7 %)	(22.1 %)	(19.6 %)	(26.2 %)		(15.6 %)	(14.0 %)	(13.8 %)	(13.2 %)	(14.3 %)	
-Wealthier	200	361	81	151	793		140	256	21	50	467		153	256	23	50	482	
	(11.1 %)	(12.5 %)	(12.0 %)	(11.8 %)	(11.9 %)		(17.8 %)	(15.7 %)	(17.2 %)	(14.8 %)	(16.2 %)		(11.8 %)	(10.0 %)	(9.3 %)	(12.5 %)	(10.7 %)	
-Wealthiest	454	1,140	201	600	2,395		185	631	50	159	1,025		328	380	110	138	956	
	(25.3 %)	(39.5 %)	(29.7 %)	(47.0 %)	(36.1 %)		(23.5 %)	(38.7 %)	(41.0 %)	(47.2 %)	(35.7 %)		(25.4 %)	(14.8 %)	(44.7 %)	(34.5 %)	(21.3 %)	

^a^Underweight (BMI < 18.5), normal weight (18.5 ≤ BMI < 25.0), and overweight (BMI ≥ 25.0)

^b^Non-agricultural jobs include the following: professional, technical, management, clerical, sales, household and domestic services, skilled or unskilled manual jobs. Agricultural jobs include self-employed as well as employed people

^*^ *p*-values were calculated using Chi-square and ANOVA tests

#### Overweight and underweight prevalence

Among OVC primary caregivers, the prevalence of overweight (Namibia: 33.4 %, Swaziland: 61.3 % and Zambia: 26.9 %) was higher than the prevalence of underweight (Namibia: 13.0 %, Swaziland: 1.7 % and Zambia: 7.4 %). In Namibia, the percentage of women who were overweight was higher for OVC primary caregivers (33.4 %), non-OVC primary caregivers (39.3 %) and non-primary caregivers not living with an OVC (30.2 %) as compared to non-primary caregivers living with an OVC (22.3 %). In Swaziland, the percentage of women who were overweight was higher for OVC primary caregivers (61.3 %) and non-OVC primary caregivers (63.7 %) as compared to non-primary caregivers living with an OVC (43.4 %) and non-primary caregivers not living with an OVC (40.9 %). In Zambia, the percentage of women who were overweight was higher for OVC primary caregivers (26.9 %) as compared to women from the other three caregiving groups (20.2 % of non-OVC primary caregivers 18.3 % of non-primary caregivers living with an OVC and 21.0 % of non-primary caregivers not living with an OVC.

### Mean BMI differences by women’s primary caregiving status within countries

**Fig. 1:**
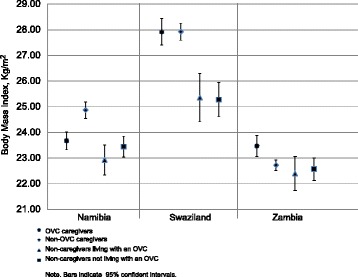
Body Mass Index by country and OVC primary caregiving status

When we compared OVC primary caregivers to the other three caregiving groups by country, we found that in Namibia OVC primary caregivers (mean BMI 23.67; 95 % Confidence Interval (CI) 23.32, 24.02) had lower mean BMI than non-OVC primary caregivers (mean BMI 24.87; 95 % CI 24.55, 25.19), (Fig. 1). The inverse situation was found in Zambia where OVC primary caregivers (mean BMI 23.47; 95 % CI 23.07, 23.88) had higher mean BMI than non-OVC primary caregivers (mean BMI 22.72; 95 % CI 22.52, 22.93). In Zambia, OVC primary caregivers had higher mean BMI than non-primary caregivers living with an OVC (mean BMI 22.40; 95 % CI 21.74, 23.06) and non-primary caregivers not living with an OVC (mean BMI 22.57; 95 % CI 22.13, 23.01) (Table 1). A similar relationship was found in Swaziland where OVC primary caregivers (mean BMI 27.92; 95 % CI 27.41, 28.44) had higher mean BMI than non-primary caregivers living with an OVC (mean BMI 25.36; 95 % CI 24.42, 26.31) and non-primary caregivers not living with an OVC (mean BMI 25.28; 95 % CI 24.61, 25.94).

When we compared OVC primary caregivers to the other three caregiving groups by country, we found that OVC primary caregivers had lower mean BMI than non-OVC primary caregivers in Namibia, and the inverse situation was found in Zambia (Fig. 1). OVC primary caregivers had higher mean BMI than non-primary caregivers living with an OVC and non-primary caregivers not living with an OVC in Swaziland and Zambia.

### Mean BMI differences by country within OVC primary caregiver status

Among OVC primary caregivers, women from Swaziland had higher mean BMI than women from Namibia and Zambia. The mean BMI and its 95 % confidence interval for OVC primary caregivers from Swaziland were in the overweight range. The mean BMI and its 95 % confidence interval for Namibian and Zambian OVC primary caregivers were in the normal weight range.

### Logistic regression models

In Namibia, OVC primary caregivers were just as likely or even less likely to be overweight than other primary caregivers. In Swaziland and Zambia, OVC primary caregivers were just as likely or more likely to be overweight than other primary caregivers. In Swaziland, OVC primary caregivers were more likely to be overweight than non-primary caregivers living with an OVC (AOR 1.56; 95 % CI 1.04, 2.34) and non-primary caregivers not living with an OVC (AOR 1.92; 95 % CI 1.46, 2.54). In Zambia, OVC primary caregivers were more likely to be overweight than non-primary caregivers living with an OVC (AOR 2.62; 95 % CI 1.80, 3.79; *p* < 0.0) and non-primary caregivers not living with an OVC (AOR 1.94; 95 % CI 1.44, 2.60), (Table 3).

**Table 3:** Association between OVC primary caregiving status and women’s overweight status^a,b^

	Swaziland ^c^ (*n* = 2,875)			Zambia ^d^ (*n* = 4,497)		
	OR	95 % CI	*p*-value	OR	95 % CI	*p*-value
	Unadjusted Models					
Model 1						
-OVC primary caregivers	0.91	(0.76–1.09)	0.30	1.44	(1.23–1.69)	<0.01
-Non-OVC primary caregivers	1.00			1.00		
Model 2						
- OVC primary caregivers	1.98	(1.35–2.92)	<0.01	1.59	(1.13–2.25)	0.01
-Non-primary caregiver living with an OVC	1.00			1.00		
Model 3						
- OVC primary caregivers	2.14	(1.65–2.77)	<0.01	1.34	(1.03–1.76)	0.03
-Non-primary caregiver not living with an OVC	1.00			1.00		
	Adjusted Models					
Model 4						
- OVC primary caregivers	0.97	(0.80–1.17)	0.72	1.16	(0.98–1.37)	0.09
-Non-OVC primary caregivers	1.00			1.00		
Model 5						
- OVC primary caregivers	1.56	(1.04–2.34)	0.03	2.62	(1.80–3.79)	<0.01
-Non-primary caregiver living with an OVC	1.00			1.00		
Model 6						
- OVC primary caregivers	1.92	(1.46–2.54)	<0.01	1.94	(1.44–2.60)	<0.01
-Non-primary caregiver not living with an OVC	1.00			1.00		

^a^Normal weight (18.5 ≤ BMI < 25.0), and overweight (BMI ≥ 25.0)

^b^Models for Namibia are not presented because an interaction term between OVC primary caregiving and age of the OVC primary caregiver was significant. Information from the stratified logistic regression modeling are presented in the “Results” section under “Effect Modification Assessment”

^c^Adjusted for age and the Absolute Wealth Index

^d^Adjusted for the number of children 5 years of age or younger in the household, women’s education and the Absolute Wealth Index

### Effect modification assessment

In Namibia, women’s age modified the effect of the association between OVC primary caregiving with overweight status. Namibian OVC primary caregivers were less likely to be overweight than non-primary caregivers not living with an OVC only among women ages 21–29 years old (AOR = 0.41; 95 % CI = 0.18–0.94) and 41–49 years old (AOR = 0.36; 95 % CI = 0.15–0.84). We did not find any other interaction effects in Namibia nor in the other two countries.

## Discussion

Findings from this study suggest that in Namibia, OVC primary caregivers were as likely, or even less likely to be overweight than women from the other primary caregiving categories. In Swaziland and Zambia, OVC primary caregivers were as likely, or even more likely to be overweight than women from the other primary caregiving categories. In other words, in these three countries OVC primary caregivers appear to have the same risks of overweight as non-OVC primary caregivers. However, OVC primary caregivers were less likely in Namibia and more in Swaziland and Zambia to be overweight than women who were not primary caregivers.

Our findings also align with the proposed African nutritional paradox that includes an increase in overweight prevalence coupled with a relatively steady underweight prevalence resulting in some households where underweight and overweight coexist [19, 20]. In this context, our descriptive analyses showed that among OVC primary caregivers, the prevalence of overweight (Namibia: 33.2 %, Swaziland: 61.3 % and Zambia: 26.9 %) was higher than the prevalence of underweight (Namibia: 13.1 %, Swaziland: 1.7 % and Zambia: 7.2 %) in these countries. Given the chronic nature of most diseases associated with overweight and the huge cost of treatment, our findings suggest that some African public health systems as well as the U.S. President’s Emergency Plan for AIDS Relief (PEPFAR) and other programs targeting OVC families should be prepared to face a new overweight epidemic alongside existing ones such as HIV/AIDS, tuberculosis, and malaria [5]. We have concluded that there is a need for revisiting current OVC strategies, their effects on OVC and OVC caregivers, and investments in Africa.

Our findings indicate that there is a lack of patterns or trends in weight status and caregiving of OVC. Strategies and investments for OVC should be tailored according to the reality of each country. Findings from logistic regression analyses also suggest that the specific characteristics of OVC primary caregivers’ nutritional status vary by country. Logistic regression analyses showed that OVC primary caregiving was associated with women’s overweight status. However, the direction of the odds ratios suggested that the role of OVC primary caregiving was a protective factor for overweight in Namibia (only among women ages 21–29 and 41–49 years old) and a risk factor for overweight in Swaziland and Zambia when compared to women from the other caregiving groups. In these three countries, socio-demographic characteristics (e.g., women’s age and education) as well as household characteristics (e.g., number of children 5 years of age or younger in the household and the Absolute Wealth Index) were potential confounders. While the mechanisms underlying OVC primary caregiving and overweight status remain unclear, further studies should analyze possible determinants that could explain differences on the direction of the odds ratios. For example, OVC primary caregivers’ household composition could influence the direction of the odds ratios. In comparison to Swaziland and Zambia, a higher percentage of OVC primary caregivers in Namibia were never married (Namibia: 41.2 %, Swaziland: 28.6 % and Zambia: 6.5 %), and/or were living without a male household member (Namibia: 70.5 %, Swaziland: 33.5 % and Zambia: 23.4 %). A study among adult females aged 18 years or more in Khayelitsha, the largest black township in Cape Town South Africa, found that being married was associated with a high BMI [21]. Researchers have also posited that the association between OVC primary caregiving and the mental health of elder caregivers may be a result of psychological distress [12]. Physiologically, chronic stress could increase cortisol and catecholamine and/or lead to unhealthy behaviors (e.g., eating poorly) that could increase caregivers’ BMI [12].

Marital status could also have influenced the direction of the odds ratio. Among OVC primary caregivers, a higher percentage of women were never married in Namibia (41.1 %) than in Swaziland (28.2 %) and Zambia (6.5 %). Namibian single women may have become OVC primary caregivers because a close family member died from AIDS. As such, Namibian OVC primary caregiver may have less social and economic support than their counterparts from Swaziland and Zambia making OVC primary caregiving status a protective factor for overweight in Namibia and a risk factor in the other two countries. In Namibia, OVC primary caregivers were less likely to be overweight than non-OVC primary caregivers and non-primary caregivers not living with an OVC. Our descriptive statistics shows that in Namibia, a higher percentage of OVC primary caregivers were not working (42.8 %) as compared to non-OVC primary caregivers (37.4 %) and non-primary caregivers not living with an OVC (37.7 %). Further studies should explore role of HIV/AIDS, health status and ability to work on the significance of the association between OVC primary caregiving and women’s overweight status. In Swaziland, non-primary caregivers were more likely to be overweight than OVC primary caregivers regardless if they were or were not living with an OVC. This association was not found with non-OVC primary caregivers. Interestingly, a higher percentage of women who were non-OVC primary caregivers (62.8 %) lived with a child less than 6 years of age as compared to non-primary caregivers living with an OVC (39.3 %) and non-primary caregivers not living with an OVC (32.6 %). Further studies should investigate if the physical and emotional work associated with being a primary caregiver for a child less than 6 years of age could have an effect on a lower probability of being overweight.

Additional studies should also explore determinants that decrease the odds for being overweight among 20–29 and 40–49 year old Namibian OVC primary caregivers as compared to non-primary caregivers not living with an OVC. Previous studies have shown differences in the magnitude of chronic energy deficiency among women at different ages revealing how BMI varies during the reproductive years [12]. A study by McGuire et al., [22] suggested that the stresses women experience during pregnancy and lactation periods could lead to considerable reduction of nutrient levels. These could be possible explanations why among 20–29 year old women, OVC primary caregivers were less likely to be overweight than non-caregivers not living with an OVC.

### Strengths and limitations

This study is unique because it included a large sample size to examine the association between reproductive age OVC primary caregiving and the primary caregivers’ BMI in communities located in Sub-Saharan Africa. Strengths of this study include the diversity and representativeness of the population of reproductive age women in three Sub-Saharan countries with different overweight prevalence. DHS surveys contain core questions that are identical across countries, and height and weight were measured rather than relying on self-reported information.

This study presents some important limitations which should be acknowledged. Women’s health status (including mental health) and dietary food intake were not available in DHS datasets. Only one nutritional measurement (BMI) was available. The cross-sectional nature of the study did not allow: 1) determining whether overweight preceded OVC’s primary caregiving or vice versa; 2) assessing whether the child might have moved to live in a wealthier household to receive care; and, 3) assessing the length of time the person was providing primary caregiving.

This study also did not measure the intensity of primary caregiving by the type or quantity of assistance provided. Self-report primary caregiver status could have been improved by providing the respondent with a more detailed description of a primary caregiver (e.g., person who is “primarily responsible for the health, safety and comfort of that child”) [23]. DHS does not collect information regarding the physical activity of the OVC primary caregiver and this study did not address their HIV status. For instance, it would be helpful to know what the mean of transportation, and distance involved, when obtaining ART or accompanying children to school. Due to different contextual factors, this study should be replicated in other African countries to further assess generalizability. It is also important to consider that missing sample analyses showed that if a respondent was from a rural area in Swaziland, a measure regarding the presence of an OVC in the household was more likely to be missing than if a respondent was from an urban area.

The World Health Organization (WHO) suggests that African countries should not just focus on addressing infectious diseases affecting their communities but should also deal with the emergence of chronic diseases [24]. Our study found that while the prevalence of overweight is on the increase in southern Africa [4], in some countries such as Swaziland and Zambia, OVC primary caregivers were more likely to be overweight as compared to non-child primary caregivers. Our results also suggest that the specific nutritional conditions of OVC primary caregivers during this time of nutritional transition in Africa differ by country. As such, it may be a better alternative to study OVC primary caregivers’ nutritional status by country instead of using pooled data from several African countries.

## Conclusion

Although programs for OVC families generally focus on undernourishment, we found that overweight also exists among OVC primary caregivers. Currently, OVC data are only available in 15 of the 44 sub-Saharan African countries where DHS is implemented. Our findings imply the need for additional nutritional studies focusing on OVC primary caregivers in other African countries to understand the nature of overweight problems among OVC primary caregivers in the entire African continent. Further studies should perform the same analysis using different definitions of child vulnerability as well as differentiate women who cared for both OVC and non-OVC. In addition, studies are needed to explore the reasons why age modified the effect of the association between OVC primary caregiving and women’s overweight status only in Namibia. In order to assess the validity of our findings, future research with additional nutritional measurements should be performed.

## Abbreviations

AOR: Adjusted Odds Ratio
BMI: Body mass index
CRUSADA: Center for Research on U.S. Latino HIV/AIDS and Drug Abuse
DHS: Demographic Health Survey
EAs: Enumeration areas
HIV/AIDS: Acquired Immune Deficiency Syndrome and the Human Immunodeficiency Virus
NIH: National Institutes of Health
NIMHD: National Institute on Minority Health and Health Disparities
OVC: Orphan and vulnerable children
PEPFAR: U.S. President’s Emergency Plan for AIDS Relief
VIF: Variance inflation factor
WHO: World Health Organization
